# Dose-Dependent Influence of RBD-Derived Amyloidogenic Peptides on SARS-CoV-2 Infectivity: A Cautionary Tale for Antiviral Design

**DOI:** 10.3390/ijms27156751

**Published:** 2026-07-28

**Authors:** Maria A. Nikiforova, Sergei Y. Grishin, Anna Y. Aksenova, Evgeniya I. Deryusheva, Ilya V. Likhachev, Roman S. Fadeev, Margarita I. Kobyakova, Alexey P. Kochetov, Alexey K. Surin, Vladimir A. Gushchin, Oxana V. Galzitskaya

**Affiliations:** 1 Gamaleya National Research Center for Epidemiology and Microbiology, 123098 Moscow, Russia; marianikiforova@inbox.ru (M.A.N.); wowaniada@yandex.ru (V.A.G.); 2 Institute of Protein Research, Russian Academy of Sciences, 142290 Pushchino, Russia; syugrishin@gmail.com (S.Y.G.); alan@vega.protres.ru (A.K.S.); 3 Laboratory of Amyloid Biology, St. Petersburg State University, 199034 St. Petersburg, Russia; a.aksenova@spbu.ru; 4 Institute for Biological Instrumentation, Federal Research Center “Pushchino Scientific Center for Biological Research of the Russian Academy of Sciences, 142290 Pushchino, Russia; janed1986@ya.ru; 5 Institute of Mathematical Problems of Biology, Russian Academy of Sciences, Branch of the Keldysh Institute of Applied Mathematics, Russian Academy of Sciences, 142290 Pushchino, Russia; ilya_lihachev@mail.ru; 6 Institute of Theoretical and Experimental Biophysics, Russian Academy of Sciences, 142290 Pushchino, Russia; fadeevrs@gmail.com (R.S.F.); kobyakovami@gmail.com (M.I.K.); 7 Baikov Institute of Metallurgy and Materials Science, Russian Academy of Sciences, Leninskiy Prospect 49, 119334 Moscow, Russia; 8 The Branch of the Institute of Bioorganic Chemistry, Russian Academy of Sciences, 142290 Pushchino, Russia; rambrent@mail.ru; 9 Pushchino Branch of the Federal State Budgetary Educational Institution of Higher Education “Russian Biotechnological University (BIOTECH University)”, Prospekt Nauki, 3, 142290 Pushchino, Russia; 10 State Research Center for Applied Microbiology and Biotechnology, 142279 Obolensk, Russia; 11 Department of Medical Genetics and Postgenomic Technologies, Federal State Autonomous Educational Institution of Higher Education I.M. Sechenov First Moscow State Medical University of the Ministry of Health of the Russian Federation (Sechenov University), 119991 Moscow, Russia; 12 Department of Virology, Faculty of Biology, Lomonosov Moscow State University, 119234 Moscow, Russia

**Keywords:** SARS-CoV-2, Spike protein, receptor-binding domain (RBD), Omicron variant, amyloidogenic properties, molecular modelling

## Abstract

The receptor-binding domain (RBD) of the SARS-CoV-2 Spike protein remains a central target for antiviral drug development. Recent in silico studies have revealed an expansion of amyloidogenic regions within the RBD of the Omicron variant, raising the possibility that amyloid-prone peptide fragments could modulate Spike function or host–virus interactions. In this study, we combined experimental assays with multiscale computational modeling to systematically characterise two short RBD-derived peptides: Pep-2 (YFPLQSYGFQ) from the ancestral Wuhan strain and Pep-3 (YFPLRSYSFR) from the Omicron BA.1 variant, the latter being predicted to have higher amyloidogenic potential. Cell-based assays demonstrated that neither peptide exhibited intrinsic cytotoxic or cytostatic effects on human lung fibroblasts or A549 lung adenocarcinoma cells at physiologically relevant concentrations, whereas significant cytotoxicity was observed in Vero E6 cells. In infection models with the B.1.1.1 (Wuhan) and BA.1 (Omicron) variants, the peptides unexpectedly enhanced virus-induced cytopathic effects at lower concentrations but inhibited viral infection at higher concentrations, indicating to a dose-dependent modulatory role for these short amyloidogenic RBD fragments. Fluorescence spectroscopy measurements did not detect the formation of stable thioflavin-T-positive amyloid fibrils. Computational analyses revealed that both peptides interact with the Spike RBD via multiple energetically favorable yet spatially heterogeneous modes, mostly outside the ACE2-binding site. Moreover, their predicted binding affinities for the ACE2 receptor were comparable, suggesting an additional route of interaction via the host receptor. Collectively, our findings demonstrate that these short amyloidogenic RBD-derived peptides exert a complex antiviral profile, with their interactions with both viral and host factors potentially shaping infection outcomes. This highlights the importance of spatially targeted and conformationally constrained peptide designs to effectively harness amyloidogenic features for antiviral therapy.

## 1. Introduction

Entry of SARS-CoV-2 into host cells is triggered by the interaction between the viral Spike glycoprotein and the human angiotensin-converting enzyme 2 (ACE2) receptor [1,2,3]. Within Spike, the receptor-binding domain (RBD) plays a pivotal role: it directly engages ACE2 and initiates the conformational changes required for membrane fusion. Given its critical function in viral entry, the ACE2-RBD interface has been widely considered a prime target for antiviral therapy [4,5]. A range of therapeutic strategies have been developed to block this interaction, including neutralizing antibodies and small-molecule inhibitors. Although highly potent, antibodies are susceptible to viral escape via RBD mutations and are also expensive to produce and distribute. Small molecules, on the other hand, often face difficulties in achieving sufficient affinity and specificity for large, relatively flat protein–protein interfaces such as ACE2-RBD complex. These limitations have motivated the exploration of alternative molecular modalities [6].

Peptide-based antivirals have emerged as an attractive intermediate strategy, combining select advantages of both small molecules and biologics [7,8]. Peptides offer modularity, tunable physicochemical properties, and the potential for rapid redesign in response to viral evolution [9]. Within this framework, short peptides derived directly from the RBD sequence have been proposed as RBD-mimetic inhibitors that can competitively block ACE2 binding. The underlying assumption of this approach is a straightforward causal chain: sequence identity or similarity to RBD residues that participate in ACE2 recognition is expected to confer binding specificity, which should consequently translate into functional inhibition [10]. Yet this assumption overlooks several crucial factors, including the three-dimensional spatial context of binding, the conformational plasticity of the Spike protein, and the possibility that energetically favorable peptide interactions may occur at non-functional or even detrimental sites [11].

Despite growing interest in RBD-derived peptides, systematic investigations addressing where these peptides actually bind on the RBD surface, and how binding location translate into functional inhibition, remain scarce. In particular, it is still unclear whether higher peptide-RBD interaction energies reliably correlate with effective blockade of the ACE2-binding interface, or whether strong binding can instead occur at regions spatially remote from the functional ACE2 epitope [12]. This question is further complicated by the rapid evolution of SARS-CoV-2 [13]. Omicron and other variants carry numerous mutations within the RBD that reshape the local interaction landscape, potentially altering both peptide binding sites and binding energetics relative to the ancestral Wuhan strain. Whether these mutations enhance, weaken, or redirect peptide-RBD interactions in a qualitative manner remains an open question with direct implications for peptide-based antiviral design [14].

Importantly, the antiviral activity of peptide-based inhibitors is frequently dose-dependent, with both efficacy and selectivity strongly influenced by peptide concentration, aggregation behavior, and the local physicochemical environment. At suboptimal concentrations, peptides may fail to achieve sufficient target occupancy on viral or host components, whereas higher concentrations can promote nonspecific interactions, self-association, or cytotoxic effects [15]. In addition, growing evidence indicates that many biologically active peptides and viral proteins contain intrinsic amyloidogenic or aggregation-prone sequences that can form supramolecular assemblies under physiological conditions [16]. Such properties can substantially affect peptide bioavailability, binding modes, and overall antiviral efficacy. Consequently, the evaluation of peptide therapeutics against SARS-CoV-2 must consider not only sequence similarity and predicted binding affinity, but also for concentration-dependent behaviour and the amyloidogenic potential of both the therapeutic peptides and their viral protein targets.

Recent studies have further suggested that, beyond classical receptor-binding mechanisms, the amyloidogenic properties of viral proteins may play a role in SARS-CoV-2 pathogenicity and host–virus crosstalk [17,18,19]. Specifically, computational predictions using FoldAmyloid [20] has revealed an expansion of amyloidogenic regions within the Spike RBD of the Omicron variant relative to the ancestral Wuhan strain [21].

**Figure 1:**
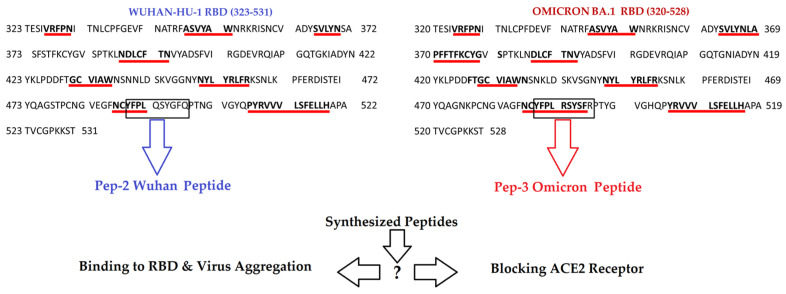
Schematic illustration of the design workflow and the proposed modes of action for peptides Pep-2 and Pep-3. The upper part depicts the amino acid sequences of the RBD from the Wuhan strain and the Omicron BA.1 variant, with amyloidogenic residues predicted by FoldAmyloid highlighted in red [20,21]. The boxed regions indicate the RBD sequence fragments from the Wuhan and Omicron variants that were selected for the synthesis of Pep-2 and Pep-3, respectively.

On the basis of these observations, we hypothesized that amyloidogenic peptide fragments derived from the Spike RBD could serve as antiviral agents against SARS-CoV-2. In contract to conventional RBD-mimetic peptides that are designed exclusively to recapitulate ACE2-binding residues, the peptides investigated in the present study were selected rationally on the basis of their predicted amyloidogenic propensity. Specifically, YFPLQSYGFQ (Pep-2) corresponds to an amyloidogenic segment identified within the Wuhan RBD, whereas YFPLRSYSFR (Pep-3) contains a longer and more pronounced amyloidogenic region predicted in the Omicron RBD. According to FoldAmyloid predictions, Pep-3 exhibits a higher amyloidogenic propensity than Pep-2, consistent with the expansion amyloid-prone region observed in the Omicron variant (Figure 1).

We proposed that such amyloidogenic peptides could exert antiviral effects through two non-mutually exclusive mechanisms. First, they may directly engage the Spike RBD, promoting co-aggregation or the formation of non-functional complexes that interfere with the conformational dynamics required for ACE2 binding. Second, the peptides may interact with host cell receptors, including ACE2, thereby reducing receptor availability and indirectly limiting viral entry.

The main objective of this study was twofold: to investigate the relationship between the predicted amyloidogenic propensity of RBD-derived peptides and their biological activity in cellular models of SARS-CoV-2 infection, and to perform a comparative analysis of peptide binding to the amyloid-specific dye ThT. In addition, we employed integrative computational approaches, including structure prediction, molecular docking, and large-scale molecular dynamics based spatial sampling. Using two short peptides with distinct amyloidogenic profiles derived, Pep-2 (derived from the Spike RBD of an ancestral Wuhan-like strain) and Pep-3 (from the Omicron variant), we aimed to determine whether differences in amyloidogenicity correlate with differences in antiviral activity, cytotoxicity, spatial binding behavior, and interaction energetics with both the RBD and ACE2.

## 2. Results

### 2.1. Dose-Dependent Antiviral Activity of Peptides and Enhancement of Cytopathic Effects

**Figure 2:**
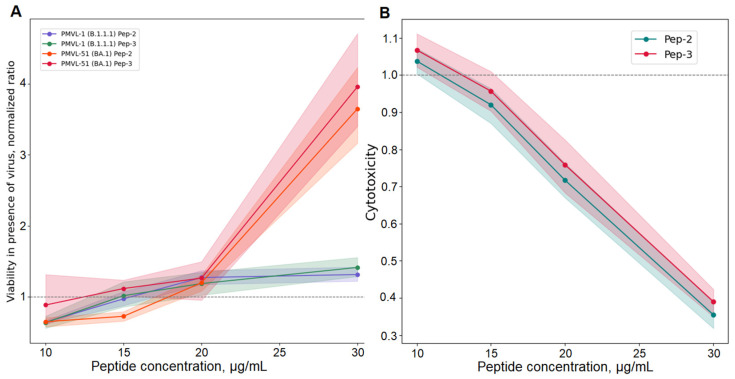
Influence of Pep-2 and Pep-3 on cytopathic effects induced by SARS-CoV-2 strains PMVL-1 (B.1.1.1) and PMVL-51 (BA.1), and their intrinsic cytotoxicity in Vero E6 cells. (**A**) Modulation of virus-induced cytopathic effects following peptide treatment at the indicated concentrations, represented as cytotoxicity-normalized fold change ratio. (**B**) Cytotoxic effects of the peptides in the absence of virus. Data are presented as mean values with bootstrap-derived 95% confidence intervals. Experiments were performed in three independent replicates (*n* = 3).

To evaluate the antiviral activity of the peptides, an MTT assay was performed on pre-seeded Vero E6 cells incubated for 4 days with 100 TCID_50_ of either PMVL-1 (B.1.1.1 Wuhan) or PMVL-51 (BA.1, Omicron) (Figure 2).

TCID_50_-based assays revealed a dose-dependent reduction in viral infectivity for both the Wuhan (B.1.1.1) and Omicron (BA.1) variants, with a more pronounced effect observed against the Omicron (BA.1) strain. Notably, pre-incubation of the viruses with the lower peptide concentration (10 μg/mL) paradoxically enhanced virus-induced cytopathic effects. This observation suggests that peptide binding at this concentration may stabilize non-neutralizing Spike conformations, potentially facilitating virus–cell interactions or promoting viral entry. However, this enhancement was lost at concentrations of 15 μg/mL and above. At this higher concentration, a clear divergence in peptide behaviour emerged against the Omicron (BA.1) variant: Pep-2 continued to promote cytopathic effect, whereas Pep-3 exerted a modest inhibitory effect. At concentrations of 20 μg/mL and higher, both Pep-2 and Pep-3 effectively inhibited infectivity of both Wuhan (B.1.1.1) and Omicron (BA.1) variants, although this inhibition was accompanied by considerable cytotoxicity (Figure 2).

### 2.2. Lack of Thioflavin T-Detectable Amyloid Aggregation by Pep-2 and Pep-3

**Figure 3:**
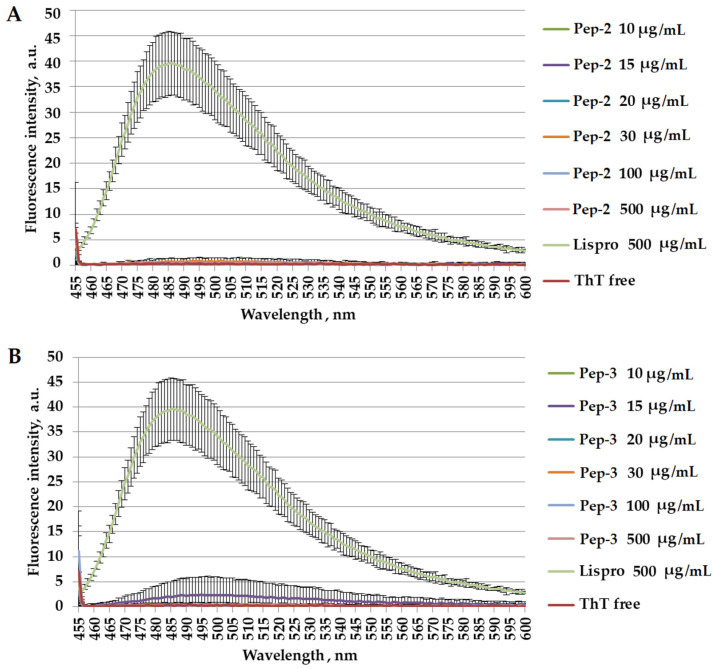
Fluorescence emission spectra of ThT alone and in the presence of Pep-2 (**A**) or Pep-3 (**B**) after 96 h of incubation at 37 °C in 50 mM Tris-HCl, 150 mM NaCl, pH 7.5, containing 10% DMSO. ThT in the absence of peptide (ThT free) served as the negative control. Preassembled amyloid fibrils of the insulin analogue lispro were used as a positive control to confirm amyloid formation and the corresponding increase in ThT fluorescence intensity. Lispro was incubation at 4000 μg/mL for 96 h at 37 °C in 20% acetic acid, pH 2.0, 150 mM NaCl, and diluted to a final concentration of 500 μg/mL at the time of measurement. Error bars indicate the standard deviation (SD) of the mean fluorescence intensity values from three independent experiments (*n* = 3).

Thioflavin T (ThT) is a widely used fluorescent probe for detecting and quantifying amyloid aggregates, as its fluorescence emission at approximately 485 nm increases markedly upon binding to fibrillar structures. Here, we examined whether the two short peptides, YFPLQSYGFQ (Pep-2) and YFPLRSYSFR (Pep-3), are capable of forming ThT-positive amyloid-like assemblies (Figure 3).

Figure 3A shows the ThT fluorescence emission spectra recorded for Pep-2 at concentrations ranging from 10 to 500 μg/mL following 96 h of incubation at 37 °C. Across all concentrations tested, no pronounced increase in ThT fluorescence intensity was observed relative to the free ThT control, indicating either the absence of amyloid-like structures capable of binding ThT, or that any structures formed by Pep-2 do not interact with the dye. Figure 3B presents the corresponding ThT fluorescence spectra for Pep-3 under identical experimental conditions and concentration ranges. Similar to Pep-2, Pep-3 did not induce a significant increase in ThT fluorescence above the negative control level.

### 2.3. Limited Cytotoxic Effects of Pep-2 and Pep-3 in Human Cell Models

**Figure 4:**
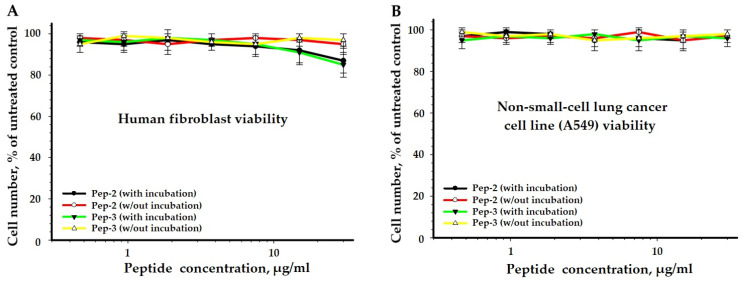
Effects of peptide treatment on the viability of human fibroblasts (**A**) and A549 lung adenocarcinoma cells (**B**). Error bars represent the standard deviation (mean ± SD); data are derived from three independent experiments.

Both peptides exhibited a favorable safety profile in the human cell-based assays (Figure 4).

No effect of peptides at concentrations up to 20 µg/mL on the viability of A549 cells was detected, which was confirmed by resazurin assay. A mild decrease in viability was observed in human lung fibroblasts, where both peptides affected viability at concentrations above 10 µg/mL. 

**Figure 5:**
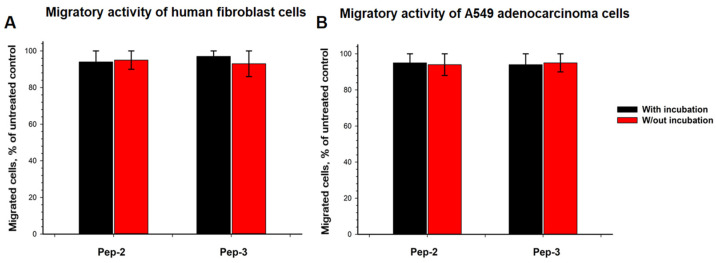
Effect of peptide treatment on the migratory activity of human lung fibroblasts (**A**) and A549 lung adenocarcinoma cells (**B**). Migratory activity is expressed as a percentage of the untreated control. Error bars represent the standard deviation (mean ± SD) from three independent experiments.

Cell migration was unaffected, indicating that the peptides do not interfere with basic cellular functions under the tested conditions (Figure 5).

Peptides at a concentration of 20 μg/mL did not affect cell migratory activity. The number of migrating fibroblasts after peptide treatment, irrespective of peptide pre-incubation, was 93 ± 7% of the control level. Similarly, the number of migrating A549 cells following peptide treatment, irrespective of peptide pre-incubation, was 94 ± 6% of the control level.

### 2.4. Global Interaction Energy Landscape from Large-Scale Molecular Dynamics Sampling

**Figure 6:**
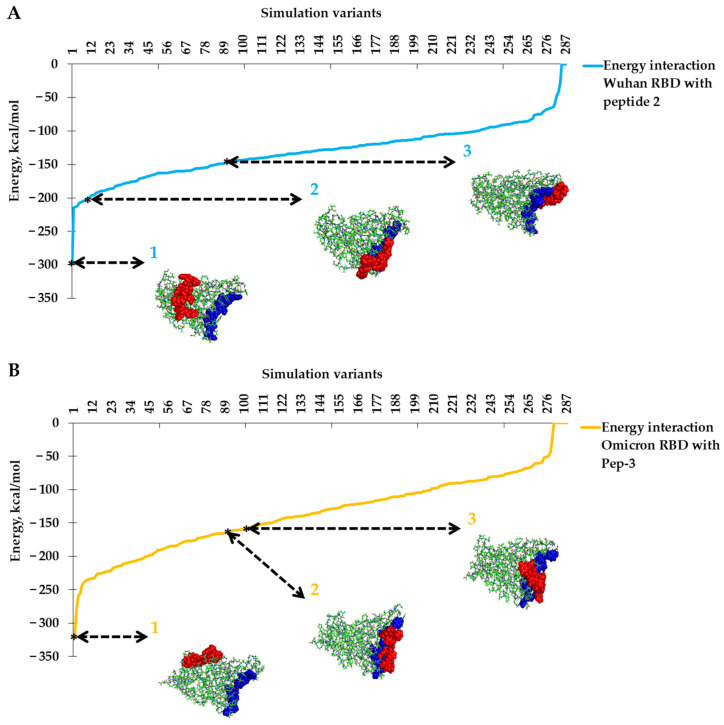
Calculated interaction energies for peptide-RBD complexes. (**A**) Wuhan RBD-Pep-2. (**B**) Omicron RBD-Pep-3. Light blue labels 1-3 indicate Pep-2 binding conformations with the Wuhan RBD, with energies of −295.7, −203.1, and −147.3 kcal/mol, respectively. Orange labels 1-3 denote Pep-3 binding conformations with the Omicron RBD, with energies of −323.5, −166.3, and −160.9 kcal/mol, respectively. Asterisk (*) indicates the calculated energy for the corresponding peptide–RBD complex. Color scheme: blue, RBD backbone; red, (Pep-2 and Pep-3) in alternative binding poses; green, other RBD regions.

To assess whether docking results adequately sampled the full spectrum of peptide-RBD interactions, we performed extensive molecular dynamics-based spatial sampling. Across all peptide-variant combinations, the interaction energies revealed a broad distribution, indicating the presence of multiple distinct binding regimes rather than a single dominant minimum. Several deep energetic minima were identified, with interaction energies reaching approximately −295 kcal/mol for the Wuhan Spike S1 and −323 kcal/mol for the Omicron Spike S1 (Figure 6).

Although the qualitative features of the interaction landscape were conserved between variants, we observed notable quantitative differences. The Omicron RBD displayed deeper energetic minima and a broader distribution of energetically favorable peptide-binding configurations than the Wuhan RBD. This broadened energy landscape suggests increased conformational and interactional plasticity in the Omicron RBD, likely arising from the accumulation of mutations that modify the local surface properties. Importantly, these variant-dependent differences did not result in preferential binding of either peptide to the ACE2-binding interface. Analysis of binding geometry further revealed a marked spatial decorrelation between interaction energy and functional relevance: the configurations with the strongest interaction energies were characterized by large center-of-mass distances and extensive contacts with RBD regions distant from the peptide’s native (original) location within the Spike protein.

### 2.5. AlphaFold 3 Predicts Distinct Peptide-Binding Sites on the RBD and ACE2 Surfaces

AlphaFold 3-based modeling of peptide-RBD complexes identified multiple potential peptide-binding regions distributed across the RBD surface for both the Wuhan and Omicron variants (Table S1). The predicted binding sites were spatially dispersed and predominantly located outside the canonical ACE2-binding motif. Notably, the overall spatial distribution of these binding regions was qualitatively conserved between the two variants, indicating that Omicron-specific RBD mutations did not generate new binding hotspots that overlap with the ACE2-binding site. For each peptide-RBD complex, five independent structural models were generated using AlphaFold 3, all of which showed consistent binding patterns.

We next employed AlphaFold 3 to model peptide interactions with the human ACE2 receptor. In contrast to the RBD complexes, both Pep-2 and Pep-3 were predicted to engage surface-exposed regions of ACE2, forming more contiguous and structurally integrated binding modes. Importantly, the peptide-binding sites predicted on ACE2 did not spatially coincide with those mapped on the RBD indicating that the two interaction surfaces are distinct and non-overlapping. Structural models of Pep-2 and Pep-3 in complex with the Wuhan and Omicron RBDs, as well as with ACE2, are provided in Supplementary Table S1.

### 2.6. Docking-Derived Binding Affinities and Variant-Dependent Trends

**Figure 7:**
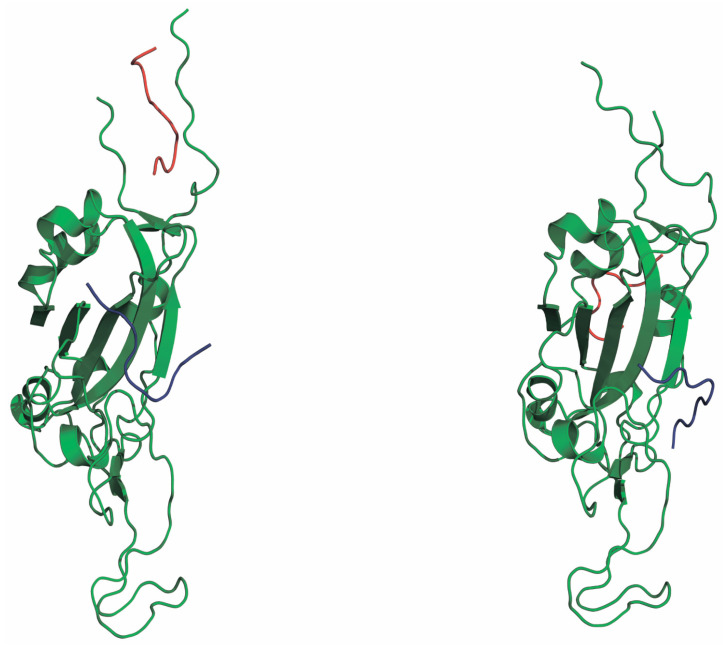
Left: Complex of Omicron RBD (green) with Pep-2 (red) and Pep-3 (blue) and Right: Complex of Wuhan RBD (green) with Pep-2 (red) and Pep-3 (blue), obtained by AutoDock Vina (https://vina.scripps.edu (accessed on 16 January 2026)).

Molecular docking using AutoDock Vina yielded moderate binding affinities for both peptides toward the RBDs of the Wuhan and Omicron variants (Figure 7).

For the Omicron RBD, Pep-2 and Pep-3 exhibited similar predicted binding free energies (−6.7 and −6.6 kcal/mol, respectively). In contrast, docking against the Wuhan RBD revealed a modest preference for Pep-3 over Pep-2, with binding energies of −7.2 and −5.7 kcal/mol, respectively. Despite these differences, no high-affinity docking poses were found to overlap significantly with the ACE2-binding interface. Instead, the top-ranked docking conformations consistently mapped to peripheral regions of the RBD, in line with the AlphaFold 3-based binding site predictions.

**Figure 8:**
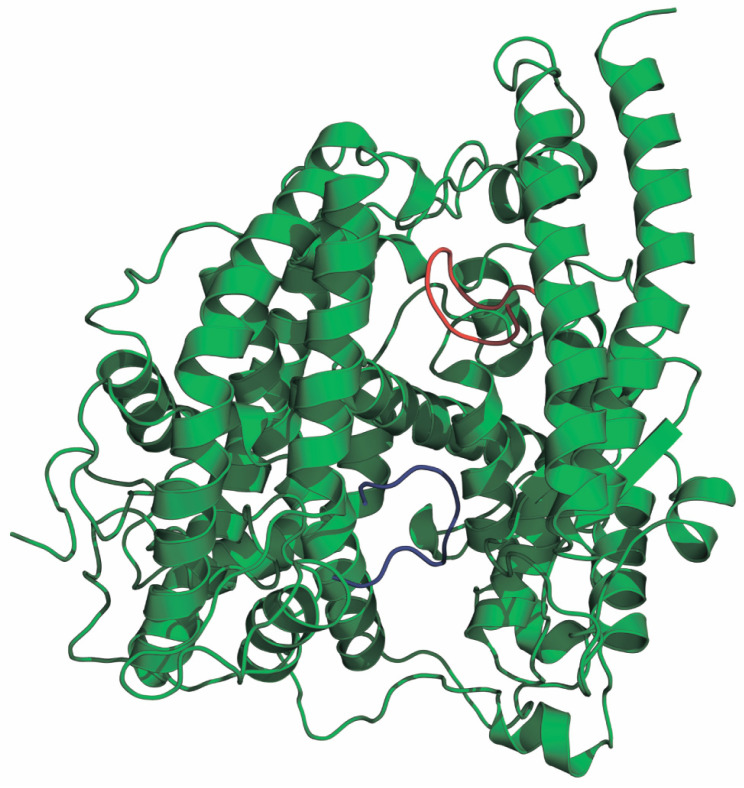
Complex of ACE2 (green) with Pep-2 (red) and Pep-3 (blue) obtained by AutoDock Vina (https://vina.scripps.edu (accessed on 16 January 2026)).

In addition, molecular docking using AutoDock Vina was carried out to estimate the binding energies between the peptides and the ACE2 receptor (Figure 8). For the ACE2-Pep-2 complex, the predicted binding free energy was −7.7 kcal/mol, which was comparable to the calculated value of −7.6 kcal/mol for the ACE2-Pep-3 complex.

## 3. Discussions

In this study, we performed an integrated experimental and computational analysis of two short peptides derived from amyloidogenic regions of the SARS-CoV-2 Spike RBD, originating from the ancestral Wuhan strain (Pep-2) and the Omicron BA.1 variant (Pep-3). The central hypothesis was that an increased amyloidogenic propensity, predicted for the Omicron-derived peptide, might translate into enhanced antiviral activity through peptide–protein co-aggregation, interference with RBD conformational dynamics, or indirect modulation of host receptors.

We observed a concentration-dependent effect of Pep-2 and Pep-3 against SARS-CoV-2 variants B.1.1.1 or BA.1 in cell-based assays. Pre-incubation of viral particles with peptides resulted in an enhancement of virus-induced cytopathic effects at lower peptide concentrations and its suppression at higher concentrations. This observation suggests that peptide binding can modulate SARS-CoV-2 virus entry and infection dynamics in a complex dose-dependent manner, which may involve interactions with both the Spike RBD and ACE2 exerting opposing effects. Notably, despite this complex interaction profile, an inhibitory effect of Pep-2 and Pep-3 was observed at concentrations of 20 μg/mL and higher, and this held true for both SARS-CoV-2 variants, reflecting the potential of these peptides for further antiviral studies.

Promotion of viral cytopathic effects is consistent with non-epitopic interactions that may alter Spike stability, surface presentation, or virus–cell contact efficiency. Similar phenomena have been reported for non-neutralizing antibodies and ligands, reinforcing the concept that functional inhibition depends critically on precise spatial targeting rather than on binding strength alone [22,23]. Alternatively, peptides may increase the effective local concentration of virions at the cell surface or partially shield the virus from non-specific inhibitory interactions in the extracellular environment. While the precise mechanism requires further investigation, this finding highlights the importance of evaluating not only neutralization but also potential infection-enhancing effects when assessing candidate antiviral peptides [24,25].

Since the present study was not designed to directly investigate the molecular mechanism underlying these observations, the following interpretations should be regarded as plausible hypotheses rather than experimentally validated mechanisms. First, peptide binding could stabilize specific conformational states of the Spike trimer. Rather than blocking the receptor-binding motif (RBM), peptides may preferentially associate with peripheral or cryptic regions of the RBD, thereby shifting the equilibrium toward conformations that favor receptor accessibility or membrane fusion competence. Stabilization of an “RBD-up” conformation, even transiently, could enhance the probability of productive ACE2 engagement. In this context, energetically favorable but spatially non-neutralizing interactions may functionally act as conformational modulators rather than inhibitors [26,27]. Second, a “bridging” or cross-linking mechanism may operate [25]. If peptides are capable of simultaneously interacting with viral and cellular surfaces, either through direct ACE2 binding or via non-specific electrostatic interactions with membrane components, they could effectively increase the local concentration of virions at the cell surface. Such a bridging effect would enhance the frequency of productive virus–cell encounters without necessarily altering intrinsic Spike–ACE2 affinity. This mechanism is conceptually analogous to previously described peptide nanofibril-mediated enhancement of viral transduction, in which aggregation-prone peptides concentrate viral particles at target membranes [28]. Third, peptides might shield virions from non-specific inhibitory interactions present in the extracellular milieu [29]. For example, transient coating of the viral surface could reduce repulsive electrostatic forces or mask destabilizing solvent interactions, thereby increasing virion stability prior to receptor engagement. Even subtle changes in virion stability can translate into measurable differences in infection efficiency under cell culture conditions. These hypotheses remain to be tested using dedicated structural and biophysical approaches.

While binding of peptides to Spike RBD may promote viral entry, engagement of host receptors such as ACE2 may represent an additional dimension of peptide activity acting in the opposing direction [30]. Computational modeling indicated comparable predicted binding energies of both peptides to ACE2. Partial receptor occupancy or conformational modulation of ACE2 could alter Spike–ACE2 interaction, influence receptor clustering and membrane distribution, or affect local microenvironmental properties in ways that indirectly modulate infection dynamics [31].

Interestingly, these findings bear conceptual similarity to antibody-dependent enhancement (ADE) [32], in which antibodies that fail to neutralize viral entry nonetheless facilitate infection through Fc receptor-mediated uptake or conformational stabilization of viral glycoproteins [33]. While the peptides studied here lack Fc domains and therefore cannot mediate classical ADE, the broader principle is analogous: binding alone does not guarantee inhibition, and under certain spatial or structural conditions, ligand engagement may paradoxically enhance infectivity. Similar effects have been described for non-neutralizing antibodies and other ligands that stabilize entry-competent conformations of viral surface proteins [34]. It should be stressed that the enhancement observed in this study was concentration-dependent and occurred in the absence of intrinsic peptide cytotoxicity, indicating that the effect is unlikely to result from compromised cell viability. Rather, it appears to reflect modulation of early entry events and RBD-ACE2 binding stoichiometries.

Taken together, the data highlight a critical design principle: both the magnitude and the spatial localization of binding must be considered simultaneously [35]. Energetically favorable interactions that do not overlap with, or allosterically disrupt, functionally essential regions may not only fail to inhibit infection but may inadvertently facilitate it. A mechanistically informed approach integrating structural localization, conformational dynamics, and functional assays is therefore essential for the rational development of peptide-based antivirals.

A notable and encouraging finding of this work is the favorable biological profile of both Pep-2 and Pep-3 in human lung fibroblasts and A549 cells. Across a range of concentrations relevant for antiviral testing, theses peptides induced only mild detectable cytotoxic or cytostatic effects at most and did not alter cell migration. This observation is particularly important in the context of peptide-based antiviral development, where non-specific membrane disruption and off-target toxicity remain common limitations. The absence of adverse cellular effects indicates that the studied peptides are well tolerated up to the concentrations at which virus inhibitory effects were observed. 

Although Pep-3 was selected based on an expanded amyloidogenic region predicted in the Omicron RBD, neither peptide formed ThT-positive amyloid-like fibrils under the experimental conditions tested. Importantly, the absence of detectable amyloid formation does not diminish the relevance of amyloidogenic properties in SARS-CoV-2 biology but instead refines their interpretation. It suggests that amyloidogenic regions may contribute to protein–protein interaction landscapes, conformational flexibility, or transient aggregation phenomena rather than to classical fibril formation when isolated from their native context. Additionally, the formation of ThT-negative amyloid-like assemblies or structurally distinct peptide aggregates cannot be excluded at this stage, since ThT selectively detects specific cross-β fibrillar architectures and may not recognize all aggregation states [36,37]. Therefore, the present findings should be interpreted as indicating the absence of detectable ThT-positive aggregates under the experimental conditions employed rather than the complete absence of peptide aggregation. Future studies employing complementary biophysical techniques, such as transmission electron microscopy, atomic force microscopy, circular dichroism spectroscopy, dynamic light scattering, Congo red binding assays, and Fourier-transform infrared spectroscopy, will be important for comprehensive characterization of the structural and aggregation properties of these peptides.

Comparison between the ancestral Wuhan-like RBD and the Omicron RBD revealed that Omicron exhibits a broader and more heterogeneous interaction energy landscape, with deeper energetic minima accessible to short peptides. This increased “plasticity” likely reflects the accumulation of mutations that reshape local surface properties and interaction patterns. However, these mutations did not create new peptide-binding hotspots within the ACE2-interacting region, nor did they enhance functional inhibition. This observation suggests that viral evolution toward immune escape and receptor optimization does not necessarily increase susceptibility to inhibition by short linear RBD-derived peptides.

From a methodological perspective, this work demonstrates that computational affinity screening must be complemented by spatial and functional filters. Global interaction energy landscapes can reveal deep minima that are misleading if considered in isolation. Incorporating binding-site specificity, geometric constraints, and functional relevance is essential for meaningful prioritization of peptide candidates. More broadly, our results support the value of publishing well-documented negative results with mechanistic insight. Such studies help refine design principles and prevent repeated exploration of strategies that are unlikely to yield functional inhibitors [38,39].

The docking-derived binding free energies, ranging from −5.7 to −7.2 kcal/mol, correspond to micromolar-scale affinities, which are unlikely to effectively compete with the high-affinity interaction between Spike RBD and ACE2. For comparison, biophysical measurements of monomeric human ACE2 binding to immobilized Omicron RBD report a dissociation constant of K_D_ = 38.9 ± 10.5 nM [40], which corresponds to an estimated binding free energy of approximately −10 kcal/mol. Computational analyses further indicate even stronger predicted binding for the Omicron BA.1.1 variant (−17.4 kcal/mol) relative to the original Wuhan strain (−12.9 kcal/mol) [41], consistent with enhanced ACE2 engagement. Collectively, these data emphasize that the physiological Spike–ACE2 interaction operates in the nanomolar regime, substantially exceeding the predicted affinities of the tested peptides. Even in cases where deeper interaction energies were observed in molecular dynamics simulations, these configurations did not correspond to ACE2-overlapping or sterically blocking binding modes. Therefore, both the magnitude and the spatial localization of binding must be considered simultaneously. Energetically favorable binding that does not overlap with, or allosterically disrupt, the ACE2-binding interface is insufficient to yield antiviral efficacy [42].

When compared to RBD interactions, docking to the ACE2 receptor yielded slightly more favorable and nearly identical binding energies for both peptides. This observation suggests that the peptides may interact with ACE2 with an affinity comparable to, or marginally higher than, their interaction with RBD, potentially providing an alternative, non-viral binding pathway. Notably, the predicted ACE2-binding sites were spatially distinct from the peptide-binding regions on the RBD, indicating independent interaction pathways. This observation suggests that host receptor engagement represents an additional dimension of peptide activity that may subtly influence infection outcomes without producing direct antiviral effects or cellular toxicity [43].

Taken together, the results of this study provide several important insights for the rational design of peptide-based antivirals. First, predicted amyloidogenicity and binding energy are insufficient criteria for functional inhibition in the absence of precise spatial overlap with critical interfaces. Second, short linear peptides derived from functional protein regions may lack the structural complexity required to reproduce native inhibitory mechanisms. Third, integrative computational approaches are essential for distinguishing energetically favorable but functionally irrelevant interactions from those with true inhibitory potential. The findings of this study suggest that effective peptide inhibitors of SARS-CoV-2 Spike protein will likely require more sophisticated designs than short linear RBD-derived sequences. Promising directions may include longer peptides spanning multiple RBM elements, conformationally constrained or cyclic peptides, multivalent constructs, or peptides designed to exert allosteric effects on Spike conformational dynamics rather than relying on direct competition with ACE2 [44,45,46].

The present study has several limitations that should be considered when interpreting the findings. A limitation of the present computational approach is that the molecular dynamics simulations were performed without explicit solvent. Although this approximation facilitates comparative analysis of peptide–protein interaction landscapes, it does not account for solvent-mediated effects, electrostatic screening, or water-dependent conformational dynamics that may influence binding energetics and complex stability. Therefore, the calculated interaction energies should be interpreted primarily in a comparative rather than quantitative manner. Future studies employing explicit-solvent molecular dynamics simulations will be valuable for validating and refining the proposed interaction models. Although the computational analyses, including molecular docking, molecular dynamics simulations, and AlphaFold 3-based structural modeling, consistently suggested potential interactions of Pep-2 and Pep-3 with both the SARS-CoV-2 Spike RBD and the ACE2 receptor, these predictions were not experimentally validated using biophysical or structural approaches. Consequently, the proposed binding modes, interaction interfaces, and mechanistic hypotheses should be regarded as plausible models rather than experimentally confirmed molecular mechanisms.

## 4. Materials and Methods

### 4.1. Peptide Synthesis and Preparation

Two short peptides derived from the RBD of the SARS-CoV-2 Spike protein were investigated. Pep-2 (YFPLQSYGFQ, 1249.4 Da) corresponds to a conserved segment of the RBD from the ancestral Wuhan strain, whereas Pep-3 (YFPLRSYSFR, 1335.5 Da) was derived from the homologous region of the Omicron variant. The peptides were synthesized by the commercial company SynPeptide (SynPeptide Co., Ltd., Shanghai, China) with a purity of >95% and were used without further modification. The correspondence between the calculated and experimentally determined peptide sequences was verified using an Orbitrap Elite mass spectrometer (Thermo Scientific, Dreieich, Germany). The experimentally determined molecular masses of the peptides matched the calculated values (Figures S1–S4).

### 4.2. Cell Culture Models and Evaluation of Virus-Induced Cytopathic Effects

Vero E6 cells were used for antiviral activity assays with SARS-CoV-2 variants B.1.1.1 and BA.1. Cells were treated with peptides at concentrations up to 30 μg/mL. Prior to the experiment, Vero E6 cells were seeded into 96-well plates (20,000 cells/well) and incubated for 18–24 h. Prepared Pep-2 and Pep-3 dilutions were mixed in an equal ratio with 100 TCID_50_ of the virus and incubated for 1 h at +37 °C. Then, the mixture was transferred into 96-well plates containing Vero E6 cells according to the layout (see below). The plates were incubated at +37 °C and 5% CO_2_ for 4 days. Next, the development of virus-induced cytopathic effect was observed, and cell viability was evaluated using the MTT assay. MTT solution (3 mg/mL) was added to the wells of the plate and incubated for 3 to 5 h at 37 °C. Following incubation, the medium was removed from the wells, and DMSO was added to each well to dissolve the crystals. OD values were read using a plate reader at 570 nm.

Since we observed a significant cytotoxicity of higher Pep-2 and Pep-3 concentrations on Vero E6 cells, we had to use a metric which accounts for it when estimating Pep-2 and Pep-3 influence on cytopathic effects induced by SARS-CoV-2 strains PMVL-1 (B.1.1.1) and PMVL-51 (BA.1). We first calculated dose-dependent effect of Pep-2 and Pep-3 in the presence of virus and absence of virus. For each condition (presence and absence of virus), raw OD measurements at each peptide concentration were first normalized by dividing by the corresponding background OD values obtained in the absence of peptides. The dose–response ratio was then defined as the normalized OD in the presence of virus divided by the normalized OD in the absence of virus, which can be defined as cytotoxicity-normalized fold-change ratio (CNFR). Confidence intervals for this ratio were estimated using nonparametric bootstrapping. Briefly, replicate measurements for each state were resampled with replacement, the normalized means were recalculated for each bootstrap sample, and the ratio between states was computed. An empirical distribution of the ratio was generated from which 95% confidence intervals were obtained using the 2.5th and 97.5th percentiles.

$$CNFR=\frac{OD\{virus+peptide\}\times OD\{mock\}}{OD\{virus\}\times OD\{peptide\}}$$

### 4.3. ThT Fluorescence Measurement

Peptide preparations Pep-2 (YFPLQSYGFQ) and Pep-3 (YFPLRSYSFR) were incubated with 200 μM ThT (Sigma-Aldrich, St. Louis, MO, USA) at 37 °C for 96 h (4 days). All experiments were performed in 50 mM Tris-HCl buffer (pH 7.5) containing 150 mM NaCl and 10% (*v*/*v*) dimethyl sulfoxide (DMSO; AppliChem, Darmstadt, Germany). ThT preparations without peptide were used as a negative control. Preparations of preformed amyloid fibrils of the insulin analog lispro were used as a positive control for amyloid formation and, accordingly, for an increase in ThT fluorescence intensity. Human insulin analog lispro (zinc-free preparation) was kindly provided by researchers from Joint-Stock Company “BIORAN Scientific Production Corporation” and the M. M. Shemyakin and Yu. A. Ovchinnikov Institute of Bioorganic Chemistry, Russian Federation. The protein was purified by the provider and used without further modification [47]. Lispro was incubated for 96 h at 37 °C in 20% acetic acid (pH 2.0) containing 150 mM NaCl. The protein concentration during incubation was 4000 μg/mL, and it was adjusted to a final concentration of 500 μg/mL prior to measurement. Fluorescence spectra were recorded using an RF-6000 spectrofluorometer (Shimadzu Corporation, Kyoto, Japan) in quartz cuvettes with an optical path length of 0.3 × 0.3 cm and a sample volume of 100 μL of peptide preparation. The excitation wavelength for ThT was 450 nm, and emission spectra were collected over the range of 455–600 nm. Measurements were performed for three independent preparations of each peptide concentration, and the mean fluorescence intensities and standard deviations were calculated.

### 4.4. Human Cell-Based Assays for Cytotoxicity and Transwell Migration

The human lung fibroblasts (HLFs) were obtained from CLS Cell Lines Service GmbH (Eppelheim, Germany). HLFs were grown in Fibroblast Growth Medium (Sigma-Aldrich, St. Louis, MO, USA) at 37 °C in a humidified atmosphere of 5% CO_2_. The human lung epithelial-like cells A549 (CCL-185) were obtained from the ATCC (Manassas, VA, USA). The A549 cells were cultured in DMEM (Sigma, St. Louis, MO, USA) supplemented with 10% fetal bovine serum (FBS) (Thermo Sci., Waltham, MA, USA) and 40 µg/mL of gentamicin sulfate (Sigma, St. Louis, MO, USA) at 37 °C in a humidified atmosphere of 5% CO_2_. The MycoFluorTM Mycoplasma Detection Kit (Thermo Sci., Waltham, MA, USA) was used to examine cell cultures for mycoplasma infection. The cell cultures did not reveal any signs of mycoplasma infection.

The cells were taken from cultures after growth for 3 days and seeded in wells of a 96-well plate in the amount of 5 × 10^3^ cells in 0,1 mL of growth medium per well. The peptides were added to cultures 24 h after cell seeding. The cell viability was assessed by the ratio of the number of living cells in the experimental and control (nontreated) cultures at 24 h after peptides addition. The number of living cells was evaluated by resazurin assay. The cells were incubated with 30 µg/mL resazurin (Sigma-Aldrich, St. Louis, MO, USA) for 4 h at 37 °C and 5% CO_2_ and then the fluorescence intensity of incubation medium was measured at Ex.532 nm/Em.590 nm using an infinite F200 plate reader (Tecan, Männedorf, Switzerland).

The effect of peptides on cell migration was analyzed using Transwell insert, 8 microns pore size (Corning, Glandale, AZ, USA). For analysis, 1 × 10^5^ cells in 0.1 mL of growth medium were seeded in the upper chamber of Transwell insert and 0.6 mL of growth medium was added in the lower chamber. Cells were cultured for 6 h; then the medium from the lower chamber was replaced with a new one, and the medium from the upper chamber was replaced with DMEM with the addition of 0.1% FBS, with or without the addition of 20 µg/mL peptides, and incubated for 24 h. The number of cells that migrated to the lower chamber was determined by staining with 0.5% crystal violet solution (Sigma, St. Louis, MO, USA).

### 4.5. Large-Scale Molecular Dynamics-Based Spatial Sampling

**Figure 9:**
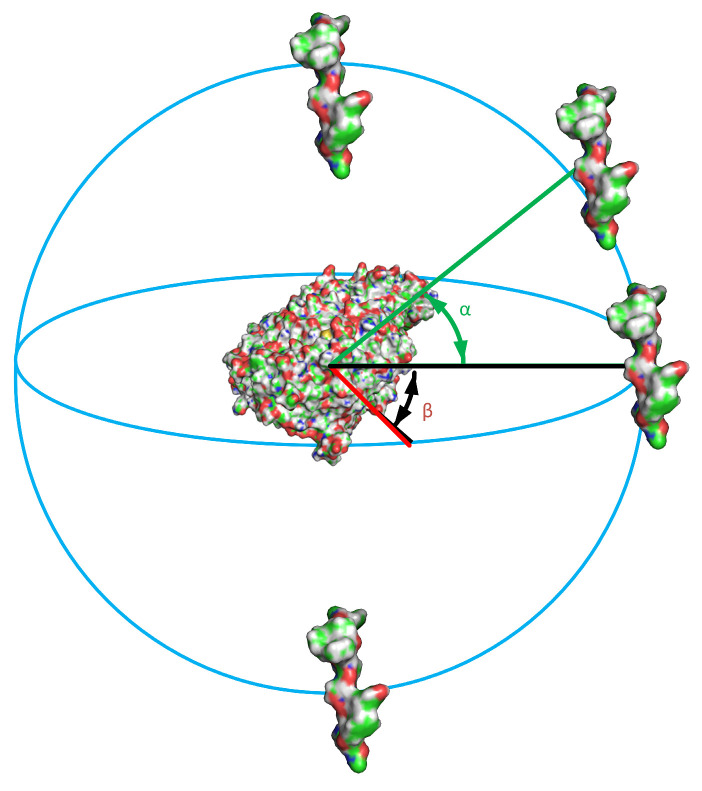
Generation of initial peptide–protein configurations by systematic angular sampling. The peptide orientation relative to the target protein was varied using spherical coordinates. Angles α and β were sampled every 30°, whereas φ assumed two orientations (0° and 180°), with ω fixed at 0°.

To explore the global interaction energy landscape beyond locally optimal docking poses, extensive molecular dynamics-based spatial sampling was performed. Simulations were carried out using the PUMA-CUDA framework in a vacuum approximation. For each peptide–RBD variant pair, a discrete combinatorial scan of peptide orientations was conducted by systematically varying three rotational angles (α, β, φ), yielding 288 initial binding configurations per variant (Figure 9).

This resulted in 288 initial configurations for each variant (576 molecular dynamics simulations in total). Each simulation was performed for 3 ns, corresponding to a cumulative simulation time of 1728 ns. Interaction energies were calculated as the sum of Coulomb and van der Waals contributions. In addition, minimal interatomic distances and center-of-mass distances between peptide and RBD were computed to characterize binding geometry and spatial proximity.

### 4.6. Identification of Peptide-Binding Sites Using AlphaFold 3

Initial peptide-RBD complexes were generated using AlphaFold 3 (https://profiles.pbcras.ru/ [48], accessed on 16 January 2026)-based modeling to explore potential peptide-binding regions on the RBD surface. Predicted complexes were analyzed to identify recurrent peptide-binding sites and to map their spatial relationship to the canonical ACE2-binding motif. Binding-site localization was assessed qualitatively and used to guide subsequent docking and molecular dynamics analyses. Contacting residues (contact distance 5 Å or less) were calculated for Cα atoms using a script in PyMOL v.2.5.0 (https://pymol.org/2/ (accessed on 16 January 2026)).

### 4.7. RBD Structural Models

Structural models of the RBD from the SARS-CoV-2 Wuhan strain and the Omicron BA.1 variant (residues approximately 320–528) were generated using AlphaFold 3 (https://profiles.pbcras.ru/ [48], accessed on 16 January 2026). For each variant, predicted structures were inspected for structural integrity and used as receptors in subsequent peptide-binding analyses. The ACE2-binding region was defined based on published structural data and used as a reference for spatial mapping of peptide-binding sites.

### 4.8. Molecular Docking

Molecular docking was performed using AutoDock Vina (https://vina.scripps.edu [49], accessed on 16 January 2026) to estimate peptide-RBD binding affinities and to refine binding poses. Pep-2 and Pep-3 were docked independently to both Wuhan and Omicron RBD models. For each peptide–RBD pair, multiple docking poses were generated, and the top-ranked conformations were selected based on predicted binding free energies. Docking results were further analyzed with respect to binding-site localization relative to the ACE2-interacting surface. Contacting residues (contact distance 5 Å or less) were calculated for Cα atoms using a script in PyMOL v.2.5.0 (https://pymol.org/2/ (accessed on 16 January 2026)).

### 4.9. Statistical Analysis

All experiments were performed with at least three independent biological replicates. Quantitative data are presented as mean ± standard deviation. Statistical analyses were performed using SigmaPlot 14 (Systat Software, San Jose, CA, USA). For normally distributed data, one-way analysis of variance (ANOVA) was used, followed by comparison of the experimental groups using Holm–Sidak test.

For the analysis of peptide effects on SARS-CoV-2 cytopathic activity, peptide-induced cytotoxicity was taken into account by calculating the cytotoxicity-normalized fold-change ratio (CNFR). Confidence intervals for CNFR were estimated using a nonparametric bootstrap approach. Replicate measurements were resampled with replacement, normalized mean values were recalculated for each bootstrap sample, and the ratio between virus-treated and virus-free conditions was computed. Empirical 95% confidence intervals were determined from the 2.5th and 97.5th percentiles of the bootstrap distribution.

## 5. Conclusions

In this study, we performed an integrated computational and experimental evaluation of two short peptides derived from the receptor-binding domain of the SARS-CoV-2. The present findings indicate that predicted amyloidogenicity and favorable docking scores alone are insufficient to identify effective SARS-CoV-2 inhibitors. In addition to appropriate spatial targeting of functionally relevant regions, successful peptide inhibitors will likely require binding affinities comparable to those of the physiological Spike–ACE2 interaction. The relatively weak predicted the docking free energies (approximately −6 to −7 kcal/mol) of the investigated peptides may therefore represent one of the factors limiting their antiviral efficacy. Our results establish a precautionary principle for the rational design of peptide inhibitors: evaluation of candidate peptides should extend beyond binding affinity measurements and neutralization assays to include mandatory assessment of their potential to enhance infection. Such comprehensive evaluation is essential to exclude unintended adverse effects and to ensure that energetically favorable or receptor-engaging interactions do not paradoxically facilitate viral entry or propagation. Future strategies aimed at exploiting amyloidogenic features of viral proteins will likely require longer constructs, conformationally constrained peptides, or multivalent designs capable of selectively targeting structurally and functionally essential regions of the Spike-ACE2 interaction axis. Overall, our findings refine the conceptual framework for RBD-derived peptide inhibitor design and emphasize the necessity of integrating structural localization, energetic analysis, and functional validation in the development of safe and effective antiviral peptides.

## Data Availability

The original contributions presented in this study are included in the article/Supplementary Materials. Further inquiries can be directed to the corresponding author.

## Supplementary Materials

The following supporting information can be downloaded at: https://www.mdpi.com/article/10.3390/ijms27156751/s1.

## Abbreviations

RBD	receptor-binding domain
ACE2	angiotensin-converting enzyme 2
