# Supplementary material for: Dose-Dependent Influence of RBD-Derived Amyloidogenic Peptides on SARS-CoV-2 Infectivity: A Cautionary Tale for Antiviral Design
Source: Int J Mol Sci. 2026 Jul 28;27(15):6751. doi: 10.3390/ijms27156751 (PMC13467299; doi:10.3390/ijms27156751)
Supplement: Supplementary file 1 [file ijms-27-06751-s001.zip › ijms-4375065-supplementary/Figure S1-S4.pdf]

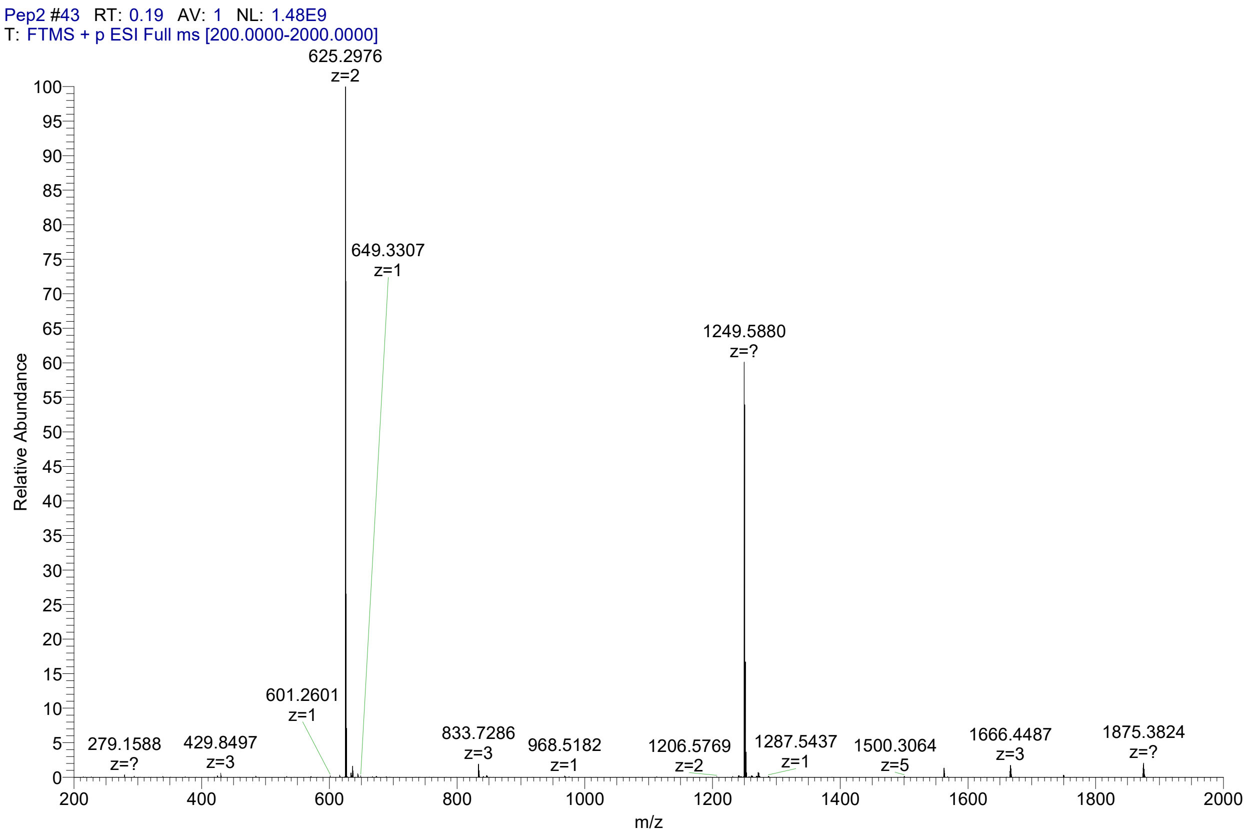

Figure S1. MS spectrm of Pep-2 (YFPLQSYGFQ). High-resolution mass spectrometry analysis of Pep-2 was performed by direct infusion of the peptide solution into an Orbitrap Elite mass spectrometer. The detected ions at *m/z* 625.2976 and *m/z* 1249.5880 correspond to the doubly charged and singly charged forms of the peptide, respectively. The experimentally determined molecular masses are in agreement with the theoretical average molecular weight of Pep-2 (1249.39 Da) calculated using the ExPASy PeptideMass tool (https://web.expasy.org/cgi-bin/peptide_mass/) for the amino acid sequence YFPLQSYGFQ.

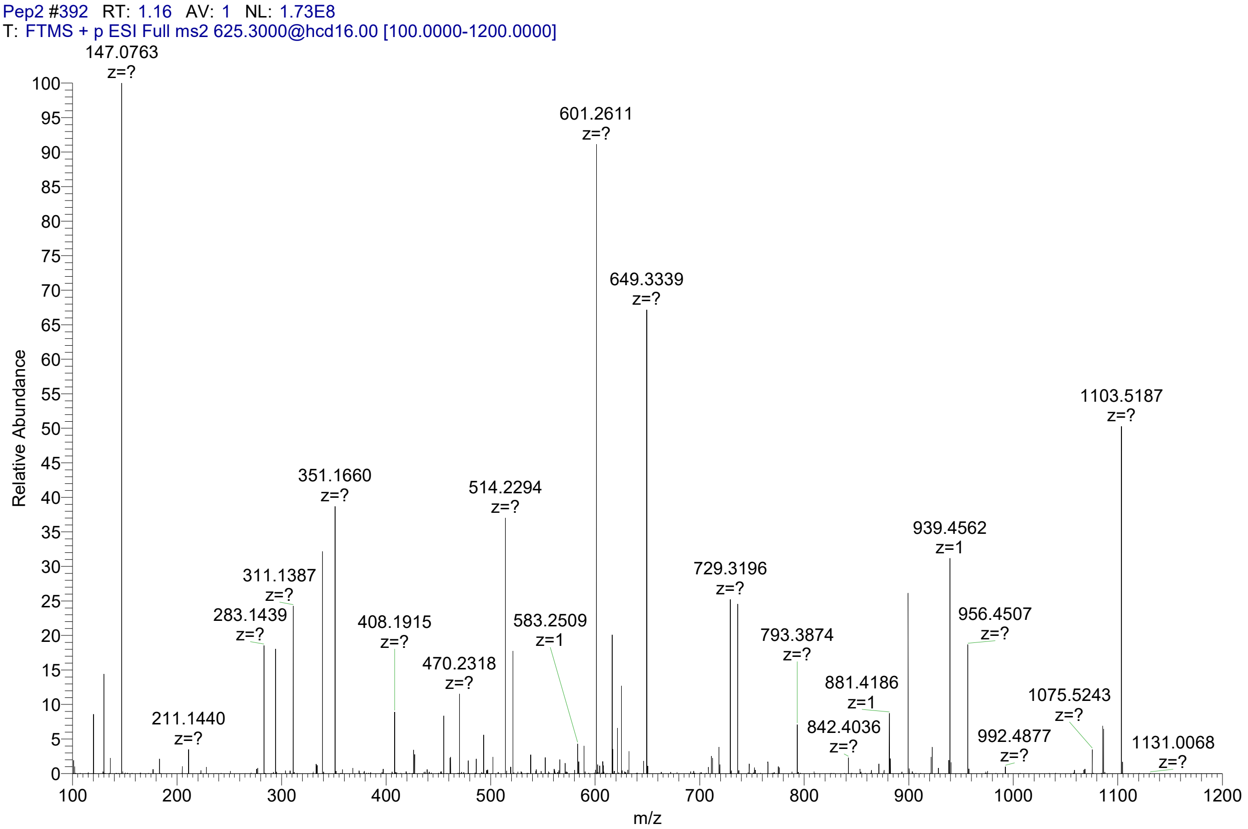

Figure S2. MS/MS fragmentation spectrum of Pep-2 (YFPLQSYGFQ).

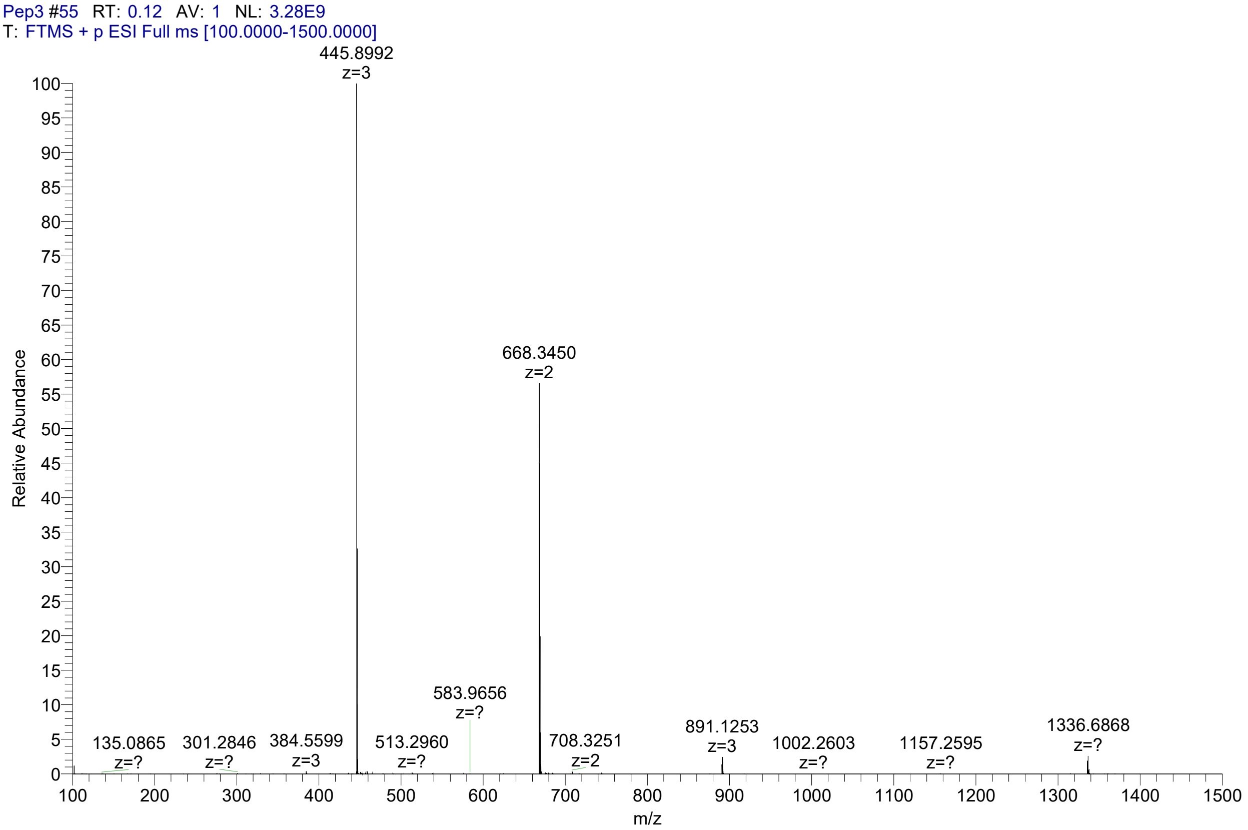

Figure S3. MS spectrum of Pep-3 (YFPLRSYSFR). High-resolution mass spectrometry analysis of Pep-3 (YFPLRSYSFR) was performed by direct infusion of the peptide solution into an Orbitrap Elite mass spectrometer. The detected ions at *m/z* 445.8992, 668.3450, and 1336.6868 correspond to different charge states of the peptide ion. The experimentally determined mass

values are in agreement with the theoretical average molecular weight of Pep-3 (1335.53 Da) calculated using the ExPASy PeptideMass tool (https://web.expasy.org/cgi-bin/peptide_mass/) for the amino acid sequence YFPLRSYSFR.

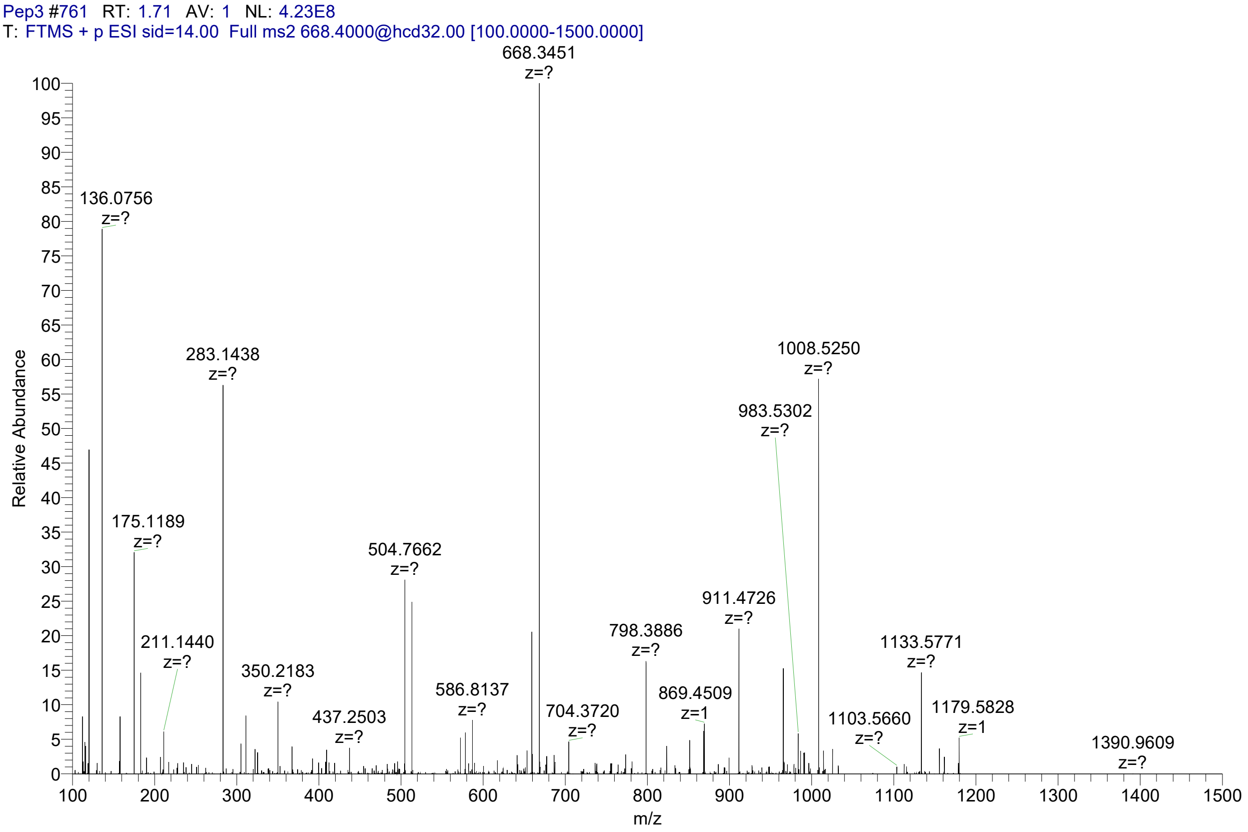

Figure S4. MS/MS fragmentation spectrum of Pep-3 (YFPLRSYSFR).
